# Behavioral Presentation, Clinical Management, and Outcome of Maternal Rejection in Mares: A Retrospective Case Series of 27 Cases

**DOI:** 10.3390/vetsci13090846

**Published:** 2026-08-22

**Authors:** Paula Nistor, Ionica Iancu, Vlad Iorgoni, Alexandru Gligor, Alexandru Ciresan, Bogdan Florea, Vlad Cocioba, Cosmin Horatiu Maris, Viorel Herman

**Affiliations:** 1 Department of Infectious Diseases and Preventive Medicine, Faculty of Veterinary Medicine, University of Life Sciences “King Mihai I” from Timişoara, 300645 Timişoara, Romania; paula.nistor@usvt.ro (P.N.); vlad.iorgoni@usvt.ro (V.I.); alexandru.gligor@usvt.ro (A.G.); viorel.herman@fmvt.ro (V.H.); 2 Department of Surgery, Faculty of Veterinary Medicine, University of Life Sciences “King Mihai I” from Timişoara, 300645 Timişoara, Romania; alexandru.ciresan@usvt.ro; 3 Department of Internal Medicine, University of Life Sciences “King Mihai I” from Timişoara, 300645 Timişoara, Romania; bogdan-alexandru.florea.fmv@usvt.ro; 4 Department of Animal Husbandry, University of Life Sciences “King Mihai I” from Timişoara, 300645 Timişoara, Romania; vlad-mihai.cocioba.fmv@usvt.ro; 5 Department of Forestry, Faculty of Engineering and Applied Technologies, University of Life Sciences “King Mihai I” from Timișoara, 300645 Timișoara, Romania; cosmin.maris@usvt.ro; 6 Academy of Romanian Scientists (AOSR), Str. Ilfov, Nr. 3, Sector 5, 050044 Bucharest, Romania

**Keywords:** maternal behavior, foal rejection, primiparous mare, oxytocin, postpartum behavior, mare–foal successful nursing acceptance, equine neonatology

## Abstract

Maternal rejection is an uncommon postpartum behavioral disorder in mares that may compromise colostrum intake, mare–foal successful nursing acceptance, and neonatal survival. This retrospective observational study described the clinical presentation, therapeutic management, and short-term outcome of 27 mares diagnosed with maternal rejection between September 2023 and June 2026 in western Romania. Data regarding breed, parity, parturition, behavioral presentation, treatment, supportive management, time to maternal acceptance, and outcome were extracted from medical records. Associations between categorical variables were evaluated using Fisher’s exact test, while differences in time to acceptance were assessed using the Mann–Whitney U test. Primiparous mares represented 59.3% of cases, and obstetrical complications occurred in 48.1%. Aggressive behavior was the predominant presentation. Primiparous mares exhibited aggressive forms of rejection significantly more frequently than multiparous mares (*p* = 0.006) and required a significantly longer interval before maternal acceptance (median: 38 h versus 21 h; U = 117.0, *p* = 0.010). Oxytocin was the most frequently administered medication, and behavioral management accompanied pharmacological treatment in most cases. Maternal acceptance was achieved in 24 of 27 mares (88.9%). Early recognition and individualized multimodal management may contribute to favorable short-term outcomes.

## Simple Summary

Maternal rejection occurs when a mare avoids, refuses to nurse, or behaves aggressively toward her newborn foal. Although uncommon, this condition can prevent the foal from receiving colostrum and may result in injury, failure of passive transfer of immunity, or death. This retrospective study describes the clinical presentation, treatment, and short-term outcome of 27 mares diagnosed with maternal rejection in western Romania. The condition ranged from avoidance and indifference to severe aggression. Primiparous mares displayed aggressive forms of rejection more frequently and required a longer period before accepting their foals than multiparous mares. Treatment consisted of individualized combinations of medications and supportive measures, including restraint, assisted nursing, temporary use of a muzzle, and supervised mare–foal contact. Maternal acceptance was achieved in 24 of the 27 mares. These findings emphasize the importance of early recognition, close supervision, and prompt intervention to protect the foal and support successful mare–foal nursing acceptance.

## 1. Introduction

Normal maternal behavior in mares develops rapidly during the immediate postpartum period and is essential for neonatal survival by ensuring successful nursing, protection of the foal, and establishment of the mare–foal bond. Shortly after parturition, the mare normally investigates the foal, permits close physical contact, and allows nursing [1]. Disruption of this process may lead to abnormal maternal behavior, including avoidance, indifference, refusal to allow suckling, fear of the foal, or overt aggression [2].

Maternal rejection is considered uncommon in horses but represents an important clinical and welfare concern. Affected foals may be unable to obtain sufficient colostrum, increasing the risk of failure of passive transfer (FPT), which predisposes them to neonatal morbidity and systemic infections [3]. Aggressive behavior may additionally result in severe traumatic injury or death if the mare–foal interaction is not closely supervised [2].

Several potential predisposing factors have been proposed, including primiparity, maternal inexperience, pain during nursing, difficult parturition, postpartum complications, altered neuroendocrine regulation, environmental stress, and individual temperament [4]. Previous reports have suggested possible breed-related and familial predispositions, particularly in Arabian mares, although these observations are based on limited clinical evidence and have not been consistently confirmed. Overall, the available literature on maternal rejection in mares remains scarce and consists predominantly of individual case reports and relatively small retrospective case series [5,6].

Management is generally based on a combination of pharmacological and behavioral interventions. Nevertheless, standardized therapeutic recommendations remain limited, and treatment is frequently adapted according to the severity of the behavior and the clinical circumstances [7].

Although maternal rejection is generally recognized as a behavioral disorder, its underlying pathophysiology remains incompletely understood. The establishment and maintenance of the mare–foal bond is a complex neuroendocrine process involving the coordinated effects of sensory stimuli, particularly olfactory, tactile, and visual cues, together with hormonal changes occurring during parturition [8]. Oxytocin is considered a key hormone involved in uterine contractions, milk ejection, and the establishment of the mare–foal bond through central neuroendocrine mechanisms. Experimental studies in several mammalian species have further demonstrated that oxytocin facilitates maternal responsiveness, whereas prostaglandins have also been implicated through their interaction with endogenous oxytocin release [9,10]. However, the relative contribution of these hormonal pathways to maternal behavior in mares remains incompletely characterized, and the mechanisms underlying maternal rejection are likely multifactorial.

Several approaches have been described to induce or restore maternal acceptance in mares affected by foal rejection or during foal grafting procedures [11]. These protocols commonly combine oxytocin with prostaglandins, sedatives or tranquilizers, whereas behavioral management includes physical restraint, supervised mare–foal interactions, assisted suckling, temporary use of restraint devices such as muzzles or hobbles, and positive reinforcement [4]. More recently, hormone-based protocols designed to promote maternal acceptance have shown encouraging clinical results while reducing the need for prolonged physical restraint [7]. However, the existing evidence remains heterogeneous and is largely derived from observational studies, limiting the development of standardized therapeutic recommendations.

Despite recent advances in therapeutic management, comprehensive clinical data describing naturally occurring maternal rejection in mares remain scarce, particularly under field conditions.

The present study was designed as a descriptive and exploratory retrospective case series. Because no comparison population of mares with normal maternal behaviour was available and treatment decisions were individualized according to clinical circumstances, the study was not designed to identify population-level risk factors or to evaluate the comparative efficacy of specific therapeutic interventions. Accordingly, the observed associations should be interpreted as hypothesis-generating findings within a clinically selected population of mares already diagnosed with maternal rejection.

Therefore, the objectives of the present retrospective study were to describe the clinical presentation, therapeutic management, and short-term outcome of mares diagnosed with maternal rejection in western Romania and to investigate potential associations between parity, parturition characteristics, behavioral presentation, treatment protocols, time to maternal acceptance, and short-term clinical outcome.

## 2. Materials and Methods

### 2.1. Study Design

This retrospective observational study included mares diagnosed with maternal rejection within the first 24 h after foaling between September 2023 and June 2026. The cases originated from equine holdings located in the western region of Romania, including Timiș, Arad, and Bihor counties. Clinical cases were managed either during ambulatory veterinary visits performed in the field or following admission to the Equine Clinic of the Faculty of Veterinary Medicine, University of Life Sciences “King Michael I” from Timișoara. Hospitalized mares included animals requiring specialized obstetrical management, such as dystocia, cesarean section, or other postpartum complications, whereas uncomplicated cases were generally treated on the farm. Overall, 27 mares fulfilled the predefined inclusion criteria and were included in the final analysis.

### 2.2. Case Selection and Clinical Evaluation

Only mares presenting maternal rejection within the first 24 h after foaling and for which complete medical records were available were included in the study. Maternal rejection was diagnosed by the attending veterinarian based on direct observation of the mare–foal interaction within the first 24 h after foaling [1,12]. For the purposes of the present retrospective analysis, behavioural findings were described primarily according to observable behavioural topographies rather than inferred affective or physiological states. Recorded behaviours included foal avoidance, defined as repeated withdrawal or movement away when approached by the foal; nursing rejection, defined as blocking udder access, moving away during udder-seeking, or otherwise preventing suckling; reduced maternal engagement, defined as the absence of investigation, close contact, or facilitation of nursing when such observations were documented; and foal-directed aggression, including threatening, chasing, attempting to bite or kick, biting, kicking, striking, or other forceful foal-directed contact [13,14].

Because the study was retrospective, the availability and specificity of behavioural descriptions varied among medical records. Terms such as “fear”, “indifference”, or other descriptions implying an underlying affective state were not interpreted as evidence of a specific behavioural mechanism unless supported by explicit clinical observations. Similarly, the term “severe aggression” was not considered an independent behavioural category unless the medical record contained sufficient information to characterize the observed behaviour.

The behavioural terminology was interpreted in accordance with established approaches to equine behavioural assessment, which emphasize the description of context-specific observable behaviours rather than the attribution of motivation or affective state [15,16]. The Video Ethogram of Equine Social Behaviour provides standardized definitions for specific equine behaviours and emphasizes the importance of contextual and temporal information when characterizing social interactions [16].

Behavioural abnormalities were classified according to the observable behavioural presentation documented in the clinical records. Recorded behavioural topographies included foal avoidance, nursing rejection, reduced maternal engagement, and foal-directed aggression. Foal-directed aggression included specific behaviours such as kicking or biting when these were documented in the clinical record. Cases in which aggression occurred together with rejection of suckling were recorded as displaying both behavioural characteristics.

The retrospective nature of the study limited the consistency and level of detail of behavioural documentation. Consequently, terms such as “fear” or “indifference” were not interpreted as evidence of a specific underlying affective state, and “severe aggression” was not treated as an independently validated severity category. No unsupported retrospective severity score was assigned when the available clinical description was insufficient. Where multiple behavioural presentations were documented, these were allowed to co-occur rather than being considered mutually exclusive categories.

For each mare, information regarding breed, parity, type of parturition, type of maternal rejection, interval between foaling and the initiation of treatment, pharmacological intervention, concomitant non-pharmacological management, time required for maternal acceptance, and final outcome was extracted from the medical records. For statistical analysis, clinical presentations were grouped into aggressive and non-aggressive forms. Aggressive forms included kicking, biting, severe aggression, and aggression associated with refusal to allow suckling [13]. Non-aggressive forms included avoidance, indifference, and nursing refusal without overt aggressive behavior.

Obstetrical complications were recorded according to the clinical diagnosis documented in the medical records. In the present cohort, complicated parturitions consisted of dystocia (*n* = 11), cesarean section (*n* = 1), and dystocia requiring subsequent cesarean section (*n* = 1). The retrospective records did not contain a standardized grading system for the clinical severity of dystocia or postpartum obstetrical complications; therefore, severity could not be reliably categorized retrospectively. For the statistical analysis, these cases were consequently grouped as complicated parturitions, while the individual clinical categories are reported descriptively to preserve the underlying clinical information.

### 2.3. Therapeutic and Supportive Managementt

Because of the retrospective design of the study, therapeutic decisions were not standardized, and the choice of pharmacological treatment was based on the attending clinician’s assessment of each individual case. Treatment selection took into consideration the type and apparent severity of maternal rejection, the degree of aggression toward the foal, the mare’s willingness or refusal to permit nursing, the need for physical restraint or sedation to allow safe mare–foal interaction, and the presence of concurrent obstetrical or postpartum complications. Oxytocin was preferentially used when restoration of maternal responsiveness and facilitation of milk let-down were considered appropriate, whereas acepromazine was used when reduction in agitation or facilitation of safe handling was considered necessary. Dinoprost tromethamine was administered according to the attending clinician’s assessment of the clinical circumstances, either alone or in combination with acepromazine. These decisions reflected routine clinical judgment rather than a predefined treatment algorithm.

Pharmacological treatment included oxytocin, acepromazine, and dinoprost tromethamine, administered either alone or in combination [17]. Pharmacological management was individualized according to the clinical presentation. Oxytocin was administered at a dose of 5–20 IU by the intramuscular or intravenous route, with repeat administration every 2–6 h when clinically indicated. Dinoprost was administered intramuscularly at 0.033–0.056 mg/kg as a single dose, with a repeat dose permitted after 24 h when necessary. Acepromazine was administered at 0.05 mg/kg by the intravenous or intramuscular route, with repeat administration every 8–12 h when clinically indicated. The timing of the first pharmacological intervention relative to parturition and the concomitant non-pharmacological measures were recorded for each case.

Supportive management consisted of behavioral interventions intended to facilitate mare–foal successful nursing acceptance and included physical restraint of the mare, assisted suckling, temporary application of a muzzle in aggressive mares, supervised mare–foal interactions, and temporary isolation when considered necessary [18]. These supportive measures were continued until the mare could safely nurse the foal or until maternal rejection was considered persistent.

### 2.4. Outcome Assessment

Treatment outcome was classified as either successful maternal acceptance or persistent maternal rejection [18]. Treatment outcome was classified as successful unrestrained nursing acceptance or persistent maternal rejection. Successful unrestrained nursing acceptance was defined as voluntary tolerance of the foal, absence of documented foal-directed aggression, and successful nursing without continuous physical restraint. This definition reflects safe nursing acceptance and does not necessarily indicate complete restoration of normal maternal behaviour or the mare–foal bond.

The interval between the first pharmacological intervention and the first documented episode of successful unrestrained nursing acceptance was recorded in hours. Cases that continued to display rejection and did not achieve unrestrained nursing acceptance during the documented clinical follow-up were classified as persistent maternal rejection [19]. Cases requiring repeated therapeutic interventions were considered successful if these criteria were ultimately achieved during the documented follow-up period. Persistent maternal rejection was recorded when the mare continued to display documented rejection behaviour or refused to permit nursing despite therapeutic intervention.

The interval between the first therapeutic intervention and the first documented episode of successful unrestrained nursing acceptance was recorded in hours. Because follow-up duration and the persistence of maternal acceptance were not prospectively standardized in the retrospective records, recurrence of rejection after the first documented successful nursing episode could not be systematically assessed [19]. Cases requiring repeated therapeutic interventions were considered successful if these criteria were ultimately achieved during follow-up. Persistent maternal rejection was recorded when the mare continued to display aggressive behavior or refused to nurse the foal despite therapeutic intervention. The interval between the first therapeutic intervention and successful maternal acceptance was recorded in hours for all successfully managed cases.

Follow-up information was obtained retrospectively through subsequent visits to the owners and/or telephone communication when available. Follow-up was not standardized or performed at predefined time points for all mares. Therefore, the absence of reported recurrence of maternal rejection should be interpreted as a finding based on the available follow-up information rather than as evidence of systematically confirmed long-term maintenance of maternal acceptance.

### 2.5. Statistical Methods

Statistical analyses were performed using IBM SPSS Statistics software 29.0.2.0 (IBM Corp., Armonk, NY, USA). Continuous variables were summarized as medians and ranges, whereas categorical variables were summarized as absolute numbers and percentages. Because of the limited sample size and the presence of several expected cell counts below five, associations between categorical variables were evaluated using Fisher’s exact test [20,21].

For statistical comparisons, parity was classified as primiparous or multiparous, and parturition was classified as uncomplicated vaginal delivery or complicated parturition, the latter including dystocia, cesarean section, and dystocia followed by cesarean section. Clinical presentation was classified as aggressive or non-aggressive. Initial pharmacological treatment was categorized according to the inclusion of oxytocin in the therapeutic protocol. Differences in the time from the first therapeutic intervention to maternal acceptance between primiparous and multiparous mares were evaluated using the Mann–Whitney U test and included only mares with successful maternal acceptance [22].

A multivariable regression analysis was not performed because the small overall sample size (*n* = 27) and the sparse distribution of observations across several categorical variables would have resulted in an unstable and potentially overfitted model. This was particularly relevant for breeds and categories of parturition and treatment, some of which contained only a small number of observations. Therefore, the analysis was intentionally restricted to predefined univariable comparisons that were considered clinically relevant and statistically appropriate for the available sample size. Potential confounding was consequently acknowledged as a limitation of the study.

All statistical tests were two-tailed, and statistical significance was established at *p* < 0.05. Because of the exploratory nature of the study and the limited sample size, no correction for multiple comparisons was applied [23].

### 2.6. Use of AI-Assisted Language Tools

AI-assisted language tools (ChatGPT) were used only to improve the English language and readability of the manuscript. All scientific content, study design, data collection, data analysis, interpretation of the results, and the final version of the manuscript were performed and approved by the authors. All data presented in the manuscript are original.

## 3. Results

### 3.1. Population Characteristics

During the study period, a total of 27 mares diagnosed with maternal rejection met the inclusion criteria and were enrolled in the study. The study population included mares from five different breeds. Primiparous mares accounted for the majority of cases (59.3%), while multiparous mares represented 40.7% of the study population. Fourteen mares (51.9%) experienced a normal vaginal delivery, whereas 13 mares (48.1%) developed obstetrical complications, including dystocia (*n* = 11), cesarean section (*n* = 1), or dystocia requiring cesarean section (*n* = 1). The baseline characteristics of the study population are summarized in Table 1.

Among the 13 mares with complicated parturition, 11 experienced dystocia, one underwent a cesarean section, and one underwent a cesarean section following dystocia. Because no standardized severity score was available in the retrospective records, these complications were not further graded according to clinical severity.

### 3.2. Clinical Presentation

Maternal rejection manifested with different behavioral patterns, ranging from indifference and avoidance of the foal to severe aggressive behavior. Overall, aggressive forms of maternal rejection, including kicking, biting, severe aggression, and aggression associated with refusal to allow suckling, accounted for more than half of all cases, whereas avoidance and complete indifference were less frequently observed.

### 3.3. Therapeutic Management

Pharmacological treatment was initiated between 6 and 24 h postpartum. Oxytocin-containing treatment was used in 14 of the 27 mares (51.9%), representing the most frequently applied pharmacological approach. Dinoprost was administered alone in six mares (22.2%) and in combination with acepromazine in two mares (7.4%), whereas acepromazine alone was administered in five mares (18.5%).

Supportive behavioral management accompanied pharmacological treatment in most mares (Table 2). Physical restraint combined with assisted suckling represented the most frequently applied supportive measure, followed by assisted suckling alone, restraint with a muzzle, and temporary isolation. Five mares received pharmacological treatment without additional supportive behavioral interventions.

### 3.4. Treatment Outcome

Maternal acceptance was successfully achieved in 24 of the 27 mares (88.9%), whereas persistent maternal rejection was recorded in three mares (11.1%). Among mares with successful outcomes, the interval between the first therapeutic intervention and maternal acceptance ranged from 12 to 72 h, with a median time of 27 h. Most successfully managed mares accepted their foals within 48 h after treatment, although one mare required a second therapeutic intervention before maternal acceptance was achieved.

All 24 mares that achieved unrestrained nursing acceptance maintained acceptance during subsequent follow-up. Follow-up information was obtained through visits to the owners and/or telephone communication, and no recurrence of maternal rejection was reported.

### 3.5. Statistical Analysis

Parity was significantly associated with the clinical presentation of maternal rejection. Primiparous mares exhibited aggressive forms of maternal rejection significantly more frequently than multiparous mares (11/16 [68.8%] vs. 2/11 [18.2%]; Fisher’s exact test, *p* = 0.0183). The odds of aggressive maternal rejection were approximately 10-fold higher in primiparous than in multiparous mares (odds ratio [OR] = 9.90, 95% confidence interval [CI]: 1.55–63.16). Among the 24 mares that successfully accepted their foals, the time from the initiation of treatment to maternal acceptance was significantly longer in primiparous mares than in multiparous mares (median: 38 h versus 21 h; Mann–Whitney U = 117.0, *p* = 0.010).

The time-to-acceptance analysis was restricted to mares that achieved documented unrestrained nursing acceptance. Therefore, this analysis evaluated differences in the recorded treatment-to-acceptance interval among successful cases and should not be interpreted as an analysis of overall treatment success or time to acceptance across the entire cohort.

Among the 24 mares that achieved documented unrestrained nursing acceptance, the recorded interval from the first pharmacological intervention to acceptance was analyzed according to parity. This analysis was conditional on eventual successful acceptance and therefore describes differences in the treatment-to-acceptance interval among successful cases rather than differences in the overall probability of acceptance.

Three mares (11.1%) did not achieve documented unrestrained nursing acceptance during the available clinical observation period. These cases included one mare displaying foal-directed biting, one mare recorded as displaying severe aggression, and one mare displaying foal-directed aggression together with rejection of suckling. The small number of persistent-rejection cases precluded reliable inferential analysis of predictors of treatment failure.

No statistically significant associations were identified between parity and treatment outcome (*p* = 0.231), type of parturition and treatment outcome (*p* = 1.000), or oxytocin-containing initial treatment and treatment outcome (*p* = 0.098). Detailed results of all statistical analyses are presented in Table 3.

## 4. Discussion

### 4.1. Clinical Characteristics of Maternal Rejection

Maternal rejection in mares is an uncommon postpartum behavioral disorder that may seriously compromise foal survival by preventing adequate colostrum intake and increasing the risk of traumatic injury [2,24]. In the present study, aggressive behavior represented the predominant clinical presentation, supporting previous reports that maternal rejection comprises a spectrum of behavioral abnormalities ranging from indifference to severe aggression requiring immediate intervention.

The relatively broad behavioral classification used in the present study represents an important consideration when interpreting the results. More detailed classifications of foal rejection have been proposed in previous clinical literature, with different underlying mechanisms potentially requiring different management strategies. However, because the present study was retrospective and the medical records did not consistently document sufficient information to distinguish fear-, pain-, or other mechanism-specific forms of rejection, such etiological classification could not be applied reliably. Future prospective studies should incorporate a standardized behavioral assessment that allows these clinically distinct presentations to be differentiated.

Primiparous mares were substantially more likely to exhibit aggressive maternal behavior than multiparous mares, with approximately 20-fold higher odds of aggressive rejection in the present cohort (OR = 20.17, 95% CI: 2.80–145.30). Although this effect estimate indicates a strong association, the wide confidence interval reflects the limited sample size and sparse distribution of observations. Therefore, the magnitude of this association should be interpreted cautiously and requires confirmation in larger prospective cohorts. The transition to normal maternal care depends on the integration of sensory stimuli and neuroendocrine changes occurring during and immediately after parturition, and disruption of these processes may delay the establishment of the mare–foal bond [25].

Although nearly half of the mares experienced obstetrical complications, no association was found between parturition type and treatment outcome. Nevertheless, postpartum pain and stress following difficult delivery may still contribute to abnormal maternal behavior and should always be considered during clinical evaluation [26].

### 4.2. Influence of Parity and Parturition on Maternal Behavior

Within the selected case series, primiparous mares were more frequently classified as displaying foal-directed aggression than multiparous mares. This finding describes a difference in behavioural presentation among mares already diagnosed with maternal rejection and should not be interpreted as evidence that primiparity is a population-level risk factor for maternal rejection. Because no comparison population of mares with normal maternal behaviour was available, the relative frequency of primiparity among all mares foaling in the source population could not be determined. Furthermore, parity should be regarded as an indicator of reproductive history rather than as a direct measure of maternal experience or a causal mechanism [13]. Experienced mares appear to recognize and respond to offspring more rapidly, whereas first-foaling mares may require a longer adaptation period before appropriate maternal behavior becomes established [27].

Primiparous mares also required significantly longer to achieve maternal acceptance following treatment than multiparous mares. This finding further supports the role of maternal experience in both the onset and recovery of maternal behavior and is consistent with learning-dependent mechanisms described in several mammalian species [28,29].

Although difficult parturition was not associated with treatment outcome, obstetrical complications remain clinically relevant because they may increase pain, fatigue, and physiological stress during the critical postpartum period. These factors should therefore be considered when evaluating mares presenting with maternal rejection [30,31,32].

### 4.3. Therapeutic Management and Clinical Outcome

Treatment of maternal rejection remains largely empirical and generally combines pharmacological therapy with behavioral management. In the present study, oxytocin-containing protocols were most frequently administered, reflecting current clinical practice. However, oxytocin is generally considered most effective when combined with supportive behavioral interventions rather than used as a sole treatment [33,34].

A recent retrospective study by Birgitsdotter et al. evaluated a defined hormone-based protocol for medical induction of maternal behaviour in 113 nurse mares and 27 mares that had rejected their own foals [7]. Although this study provides important contemporary evidence regarding hormonal management, its population and outcome framework differ from those of the present investigation. The study evaluated a standardized foal-grafting protocol, whereas treatment in the present cohort was individualized according to clinical circumstances. In addition, the outcome construct and treatment setting differed between the studies. Therefore, the results of Birgitsdotter et al. should be considered complementary evidence rather than directly comparable treatment-efficacy data for the present case series.

The present study adds clinical observations from naturally occurring maternal rejection managed under routine field and hospital conditions, but it does not overcome the methodological limitations inherent to the existing observational evidence. As a retrospective case series without a comparison group and with non-standardized treatment allocation, the study can describe treatment patterns and short-term outcomes but cannot establish causal relationships or determine the comparative efficacy of individual interventions.

No significant association was identified between oxytocin-containing initial protocols and treatment outcome. However, this finding should not be interpreted as evidence that the evaluated treatment protocols have equivalent therapeutic efficacy. Treatment allocation was not randomized or standardized but was determined by the attending clinician according to the clinical circumstances of each case. Consequently, differences in disease severity, behavioral presentation, concurrent obstetrical complications, or other unmeasured clinical factors may have influenced both treatment selection and outcome. The small sample size further limits the statistical power to detect clinically relevant differences between treatment approaches. Therefore, the present findings should be interpreted as descriptive observations of treatment use and short-term outcome in routine clinical practice rather than as comparative evidence of therapeutic efficacy.

Supportive management, including physical restraint, assisted suckling, supervised mare–foal interactions, and temporary use of muzzles when necessary, accompanied pharmacological treatment in most mares [2]. Maternal acceptance was subsequently recorded in most cases while mares were receiving heterogeneous pharmacological and supportive management. Because there was no untreated comparison group and treatment allocation was neither randomized nor standardized, the observed outcomes cannot be attributed to treatment timing, any individual intervention, or multimodal management. The findings, therefore, describe management patterns and short-term outcomes under routine clinical conditions rather than comparative treatment efficacy.

### 4.4. Clinical Implications

Maternal rejection should be regarded as a neonatal emergency because delayed nursing increases the risk of failure of passive transfer and subsequent infectious diseases. In addition, aggressive maternal behavior may cause severe traumatic injuries to the foal, emphasizing the need for continuous supervision until normal maternal behavior is restored [34].

The favorable outcome observed in most mares indicates that early recognition and prompt intervention can successfully restore maternal acceptance in the majority of cases. Particular attention may be warranted when monitoring primiparous mares that present with maternal rejection, as primiparous mares in the present case series were more frequently classified as displaying foal-directed aggression and required a longer recorded interval to nursing acceptance among successful cases. These findings should not be interpreted as evidence that primiparity increases the population-level risk of maternal rejection [2].

### 4.5. Study Limitations and Future Perspectives

The retrospective design, relatively small sample size, and lack of standardized behavioral scoring represent the principal limitations of the present study. In addition, endocrine parameters potentially involved in maternal behavior were not evaluated, preventing assessment of the physiological mechanisms underlying maternal rejection.

The absence of standardized behavioural observations also prevented a reliable exploratory analysis of behavioural severity in relation to time to maternal acceptance or persistent rejection. Although such an analysis could have direct clinical relevance, retrospective assignment of severity grades based on inconsistent narrative descriptions would have introduced substantial classification bias. Future prospective studies should therefore record behavioural topographies, their sequence and duration, and clinically relevant severity indicators using predefined criteria, allowing their relationship with maternal acceptance and foal outcomes to be evaluated.

In addition, treatment allocation was based on individual clinician judgment rather than a predefined therapeutic algorithm, and the retrospective records did not allow the relative contribution of specific clinical factors to treatment selection to be quantified. This introduces a potential for treatment-selection bias and limits direct comparison of the observed outcomes between pharmacological protocols.

The absence of multivariable modeling also limited the ability to adjust simultaneously for potential confounding factors such as parity, breed, type of parturition, and treatment protocol.

Follow-up after maternal acceptance was retrospective and was not performed according to a standardized schedule for all mares. Consequently, the absence of reported recurrence should not be interpreted as evidence of systematically confirmed long-term maternal acceptance.

Despite these limitations, this study provides valuable clinical information on naturally occurring maternal rejection managed under routine field and hospital conditions. Future prospective multicenter studies using standardized treatment protocols, objective behavioral assessment, and endocrine profiling are warranted to improve understanding of this condition and optimize evidence-based therapeutic recommendations.

## 5. Conclusions

Within this retrospective case series, most mares subsequently achieved unrestrained nursing acceptance while receiving heterogeneous pharmacological and supportive management. Primiparous mares within the selected case population were more frequently classified as displaying foal-directed aggression and, among mares achieving acceptance, had longer recorded treatment-to-acceptance intervals than multiparous mares. However, the absence of a comparison population and the non-standardized allocation of treatment preclude causal interpretation and evaluation of treatment efficacy. The study therefore provides descriptive, hypothesis-generating clinical information rather than evidence of population-level risk factors or comparative treatment effectiveness. Future prospective studies should use standardized behavioural definitions, predefined treatment protocols, standardized follow-up, and systematic assessment of foal outcomes to clarify the clinical determinants and management of maternal rejection in mares.

## Figures and Tables

**Table 1:** Baseline characteristics of the 27 mares included in the study.

Characteristic	n (%)
Total mares	27
Parity	
Primiparous	16 (59.3)
Multiparous	11 (40.7)
Breed/type	
Pony	7 (25.9)
Warmblood	6 (22.2)
Thoroughbred	6 (22.2)
Draft	5 (18.5)
Arabian	3 (11.1)
Type of parturition	
Normal vaginal delivery	14 (51.9)
Dystocia	11 (40.7)
Cesarean section	1 (3.7)
Dystocia followed by cesarean section	1 (3.7)

**Table 2:** Pharmacological treatment and supportive management used in the 27 mares.

Intervention	n (%)
Pharmacological treatment	
Oxytocin	14 (51.9)
Dinoprost	6 (22.2)
Acepromazine	5 (18.5)
Dinoprost + Acepromazine	2 (7.4)
Supportive management	
Restraint + assisted suckling	6 (22.2)
Assisted suckling	5 (18.5)
Restraint	4 (14.8)
Restraint + muzzle	4 (14.8)
Restraint + isolation	3 (11.1)
None	5 (18.5)

**Table 3:** Summary of statistical analyses.

Comparison	Statistical Test	*p* Value
Parity vs. aggressive/non-aggressive clinical presentation	Fisher’s exact test	0.006
Parity vs. time to maternal acceptance	Mann–Whitney U test	0.010
Parity vs. treatment outcome	Fisher’s exact test	0.231
Parturition type vs. treatment outcome	Fisher’s exact test	1.000
Oxytocin-containing vs. non-oxytocin initial treatment and outcome	Fisher’s exact test	0.098

## Data Availability

The data presented in this study are available from the corresponding author upon reasonable request. The data are not publicly available because they contain information derived from privately owned clinical cases.
